# Nutritional Qualities, Metabolite Contents, and Antioxidant Capacities of Yardlong Beans (*Vigna unguiculata* subsp. *sesquipedalis*) of Different Pod and Seed Colors

**DOI:** 10.3390/antiox13091134

**Published:** 2024-09-19

**Authors:** Yu-Mi Choi, Myoung-Jae Shin, Hyemyeong Yoon, Sukyeung Lee, Jungyoon Yi, Xiaohan Wang, Kebede Taye Desta

**Affiliations:** 1 National Agrobiodiversity Center, National Institute of Agricultural Sciences, Rural Development Administration, Jeonju 54874, Republic of Korea; cym0421@korea.kr (Y.-M.C.);; 2 International Technology Cooperation Center, Technology Cooperation Bureau, Rural Development Administration, Jeonju 54875, Republic of Korea; 3 Institute of Crop Sciences, Chinese Academy of Agricultural Sciences, Beijing 100081, China

**Keywords:** antioxidants, asparagus bean, diversity, legume, metabolomics, nutrition, vegetative cowpea

## Abstract

Studying the effects of genetic and environmental factors on plant biochemical components helps in selecting the best varieties for the food industry and breeding programs. This study analyzed the nutritional qualities, secondary metabolites, and antioxidant activities of 14 field-grown yardlong beans accessions and how they are affected by differences in pod and seed colors. The analyzed parameters varied significantly among the yardlong bean accessions, with variances ranging from 1.36% in total unsaturated fatty acid content to 51.01% in DPPH^•^ scavenging activity. Accessions YLB4, YLB7, and YLB14 performed the best, showing antioxidant indices of 100.00, 70.10, and 67.88%, respectively. Among these, YLB14 showed a characteristic property, having the highest levels of vitamin C (2.62 mg/g) and omega-6 to omega-3 ratio (2.67). It also had the second highest dietary fiber (21.45%), stearic acid (4.44%), and linoleic acid (40.39%) contents, as well as the lowest thrombogenicity index (0.38). Although cluster and principal component analyses did not clearly separate the yardlong beans based on pod or seed color, analysis of variance revealed that these factors and their interaction had significant effects on total phenol, DPPH^•^ scavenging activity, ABTS^•+^ scavenging activity, and reducing power. In contrast, the nutritional parameters, except for dietary fiber, were not significantly affected by pod and seed color variations. Therefore, consuming yardlong beans of different pod and seed colors may not affect the overall nutrient intake. In general, this study identified yardlong beans with green pods and black seeds as good sources of antioxidants. Accordingly, further metabolomics and genomics studies are suggested to thoroughly explore their characteristics.

## 1. Introduction

Yardlong bean (*Vigna unguiculata* subsp. *sesquipedalis*), also called asparagus bean, is a type of legume plant that belongs to the Fabaceae family. It is a sub-species of cowpea and is commonly known as vegetative cowpea [1,2]. This crop is usually cultivated in tropical and subtropical regions, either alone or as part of intercropping and sequential cropping systems. However, challenges, such as the lack of improved varieties, limited extension services, low market prices, and susceptibility to pests and diseases, continue to affect its production [1].

Yardlong bean is characterized by its long pods that grow in a climbing manner. The immature pods and seeds of yardlong beans are widely consumed in Southeast Asian countries [3,4]. Various studies have emphasized the nutritional value of yardlong bean pods and seeds, highlighting their richness in essential nutrients, protein, fiber, vitamins, and polyphenols [5,6,7,8]. These metabolites provide numerous health benefits, such as boosting immunity, reducing the risk of chronic diseases, regulating blood glucose level, and serving as antioxidants [5,9,10].

Several factors affect the overall quality of crops, including their agronomic characteristics, nutrient levels, and phytochemical compositions. These factors include variations in genetic backgrounds, farming conditions, abiotic stresses, and adverse weather conditions resulting from climate change [11,12,13,14]. Accordingly, developing improved crop varieties that can withstand these challenges has become a primary objective in breeding. Thus far, breeding programs for yardlong beans have primarily focused on enhancing yield by improving specific traits such as pod quantity per plant, early maturation, and adaptability to diverse agroclimatic conditions, among others [1,15,16,17]. Recently, such studies in the United States of America have resulted in the registration of four yardlong bean germplasms that are resistant to pests [18].

Understanding how genetic and environmental factors affect the biochemical compositions of crops is as crucial as enhancing yield traits and field performances. Having a deep understanding in this regard is essential for improving the nutritional value of crops and optimizing their health advantages, as well as increasing their use in food industries [11]. Accordingly, several studies have investigated how nutritional components and health-promoting secondary metabolites in various crops, such as legumes, are influenced by agricultural traits, environmental factors, and genetic factors [11,13,19]. Nonetheless, existing studies on yardlong beans lack comprehensive investigation in these regards [13,20]. Previously, a study conducted a decade ago attempted to compare the distribution and levels of specific anthocyanins in the pods and seeds of yardlong beans [21]. In another study, Flyman and Afolaya [22] studied how the maturity stage affects mineral contents in the leaves of yardlong beans. Apart from these studies, there is a lack of research focusing on investigating the effects of other factors on the biochemical compositions of yardlong beans. 

Our gene bank at the National Agrobiodiversity Center, Rural Development Administration (Jeonju, Republic of Korea), contains approximately 227 yardlong bean genetic materials thus far. Our study aims to fill the research gaps described before utilizing these genetic materials. Recently, the effects of origin and genotype variations on the levels of several biochemical components have been explored [23]. As part of an ongoing study, this study aims to provide a comprehensive analysis regarding the variations in major nutritional components, secondary metabolite contents, and antioxidant activities across 14 field-grown yardlong bean accessions. Moreover, like other legumes, yardlong beans are found in different seed and pod colors, which are controlled by different genes and, hence, could affect their overall biochemical compositions [15,24,25,26]. This study also statistically analyzed the effects of immature pod color and seed color variations on the levels of the analyzed biochemical parameters for the first time. This study could help to enhance our understanding of the biochemical diversity present in yardlong beans. Moreover, the results of this study could highlight the associations between pod and seed colors with health-promoting metabolites. This information could be utilized in food industries to select superior varieties and in breeding programs aimed at developing cultivars with improved qualities.

## 2. Materials and Methods

### 2.1. Chemicals and Reagents

All the chemicals and reagents used in this study were analytical grade. Ethanol and sulfuric acid were ordered from Fisher Scientific (Pittsburgh, PA, USA), while the remaining chemicals and reagents were purchased from Sigma-Aldrich (St. Louis, MO, USA).

### 2.2. Plant Material Collection and Sample Preparation

Samples of the 14 yardlong bean accessions (Table S1) were obtained from the 2022 growing season conducted at the National Agrobiodiversity Center, Rural Development Administration (Jeonju, Republic of Korea). The immature pods were collected when fully colored (green or light green), freeze-dried in an LP500 freeze dryer (ilShinBioBase, Dongducheon, Republic of Korea), pulverized, and examined for the contents of vitamin C, secondary metabolites, and antioxidant activities. Similarly, the matured seeds (brown or black) were also hand-harvested, freeze-dried, pulverized, and assessed for their nutritional contents and fatty acid levels. Sample preparation for the analysis of vitamin C, fatty acids, secondary metabolites, and antioxidant activities was conducted according to a recently reported method without any modifications [23]. All powdered samples and extracts were stored at −20 °C when not used.

### 2.3. Analysis of Nutritional Components

The determination of nutritional components, including crude protein, total fat, dietary fiber, and crude fiber contents, was achieved using standard methods recommended by the Association of Official Analytical Chemists (AOAC), as detailed in our recent study [23,27]. Briefly, the total protein content was determined using the Kjeldahl method, while the total fat content was estimated using the Soxhlet method. Crude fiber and dietary fiber levels were examined using Fiber Analyzer and Analytical Fibertec instruments (FOSS, Hillerød, Denmark), respectively. The results of the nutritional components were reported as a percentage based on the weight of the dried sample from triplicate measurements. The content of vitamin C was analyzed by a 1260-Infinity Quaternary High-performance Liquid Chromatography system (Agilent Technologies, Santa Clara, CA, USA). The column used was a Zorbax SB-18 (4.6 × 250 mm, 5 µm) which was maintained at a temperature of 30 °C. A binary solvent composed of water with 0.1% trifluoroacetic acid (A) and methanol (B) was used as a mobile phase. The gradient condition started with 5% B and increased to 50% over 10 min. Then, the flow was returned to 5%B over another 10 min. The post-run time was set at 5 min. The acquired chromatogram was monitored at 245 nm using a diode-array detector (DAD), and the level of vitamin C (in mg/g) was determined using *L*-ascorbic acid as a reference standard. The analysis of fatty acids was carried out using a QP2010 gas chromatography–flame ionization detector instrument (Shimadzu, Kyoto, Japan). The column was an HP-INNOWAX (30 m × 0.250 mm, 0.25 µm). The temperature gradient was initially set at 100 °C and gradually increased to 170 °C at a rate of 60 °C/min. After a 1 min hold time, the temperature was raised to 240 °C at a 6.5 °C/min ramp and held for another 1 min. Identification of the fatty acids was achieved by comparing retention times with external standards. The content of each fatty acid was determined as peak area percentages from triplicate measurements. The lipid quality indices including the atherogenicity index (IA) and the index of thrombogenicity (IT) were determined using Equations (1) and (2), as previously outlined [28,29]. In each equation, PUFA represents the total polyunsaturated fatty acid content, and MUFA represents the total monounsaturated fatty acid content.

(1) $$IA=\frac{C12:0+4\times C14:0+C16:0}{\sum MUFA+\sum PUFA\;n-6+\sum PUFA\;n-3}$$

(2) $$IT=\frac{C14:0+C16:0+C18:0}{0.5\sum MUFA+0.5\sum PUFA\;n-6+3\sum PUFA\;n-3+\frac{\sum PUFA\;n-3}{\sum PUFA\;n-6}}$$

### 2.4. Analysis of Total Metabolite Contents

The total phenolic (TPC), total tannin (TTC), and total saponin (TSC) contents were determined using known colorimetric assays, as detailed in our recently reported study [23]. Briefly, TPC was determined by the Folin-Ciocalteu technique and expressed in milligrams of gallic acid equivalents per gram of dried sample weight (mg GAE/g), with gallic acid as the reference standard. TTC was measured using the vanillin-HCl method and reported as milligrams of catechin equivalents per gram of dried sample weight (mg CE/g) using catechin as the standard. Similarly, TSC was determined using the vanillin-sulfuric acid assay and calculated as milligrams of diosgenin equivalent per gram of dried sample (mg DE/g) with diosgenin as the standard. Absorbance was measured using an Eon Microplate Spectrophotometer (Bio-Tek, Winooski, VT, USA) for each analysis.

### 2.5. Analysis of Antioxidant Capacities

The antioxidant capacities of each yardlong bean accession were assessed by three independent colorimetric assays [23]. Overall, the DPPH^•^ scavenging activity and reducing power (RP) were determined in milligrams of ascorbic acid equivalents per gram of dried sample weight (mg AAE/g) using ascorbic acid as a standard. ABTS^•+^ scavenging activity was determined in milligrams of Trolox equivalents per gram of dried sample weight (mg TE/g) using Trolox as a standard. During each assay, the antioxidant activity of the yardlong bean accessions was estimated using the following equation (Equation (3)).

(3) $$Antioxidant\;capacity\left(mg\;STD\;g^{-1}DW\right)=\frac{\left(C\times V\right)}{m}\times Df$$

where C is the concentration (mg/mL) of the sample corresponding to the calibration curve of the standard (STD) used, V is the sample volume (mL), Df is the dilution factor, and m is the sample weight (g).

### 2.6. Antioxidant Index

The antioxidant index (AI) was determined using the six different colorimetric assays, including DPPH^•^ scavenging activity, ABTS^•+^ scavenging activity, RP, TTC, TSC, and TPC according to a previously reported method [30]. In each assay, the highest value was considered 100%, and the remaining lower values were converted to numerical scales using the following equation (Equation (4)). Then, the AI of each accession was calculated as the average relative percentage value of the converted scales.

(4) $$AI=\frac{Sample\;score}{Best\;score}\times 100$$

### 2.7. Statistical Analysis

In this study, the results are presented as the mean ± standard deviation (SD) from triplicate measurements. Statistical analysis was performed using analysis of variance (ANOVA) followed by Duncan’s multiple range test at a significance level of *p* < 0.05 using XLSTAT software version 2019.2.2 (Lumivero, CO, USA). Hierarchical cluster and Pearson’s correlation analyses were computed using R-software (version 4.0.2, R-project), while principal component analysis was carried out using JMP-Pro software version-17 (SAS Inc., Cary, NC, USA).

## 3. Results

### 3.1. Nutritional Components

#### 3.1.1. Crude Protein, Total Fat, Crude Fiber, and Dietary Fiber Contents

**Figure 1:**
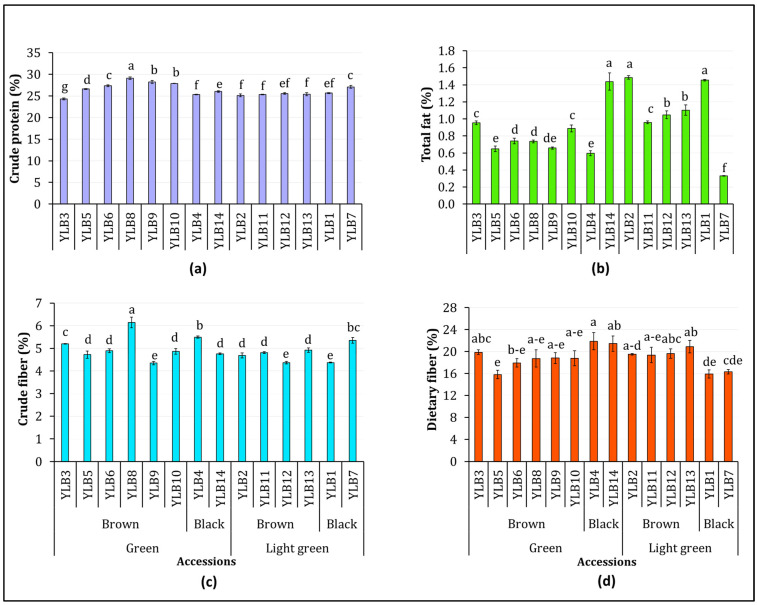
The contents of crude protein (**a**), total fat (**b**), crude fiber (**c**), and dietary fiber (**d**) across 14 yardlong bean accessions of different immature pod colors (green and light green) and seed colors (brown and black). Different letters on bars (charts) indicate significantly different means at *p* < 0.05.

The contents of crude protein, total fat, crude fiber, and dietary fiber in each of the 14 yardlong bean accessions are shown in Figure 1. The crude protein content ranged from 24.31 to 29.10%, with an average of 26.36%, indicating the least variance (5.05%) compared to other components (Figure 1a). In contrast, the total fat content ranged from 0.33 to 1.49% with an average of 0.93%, showing the highest variance (36.12%) (Figure 1b). With means of 4.92% and 18.93%, both crude fiber (4.35–6.14%) and dietary fiber (15.83–21.90%) contents showed comparable variances of 9.65% and 9.74%, respectively (Figure 1c and Figure 1d). Accession YLB8 had the highest levels of crude protein and crude fiber, both significantly differing from the other accessions (*p* < 0.05). Conversely, accessions YLB3 and YLB9 had the lowest levels of crude protein and crude fiber, respectively. Accessions YLB2 and YLB7 had the highest and lowest fat contents, while YLB4 and YLB5 had the highest and lowest dietary fiber contents, respectively. The highest fat content found in accession YLB2 was significantly different from all other accessions, except YLB1 and YLB14, which had the second highest (1.45%) and third highest (1.44%) fat contents, respectively (*p* < 0.05). Similarly, the highest dietary fiber content in accession YLB4 was significantly different from accessions YLB1, YLB5, YLB6, and YLB7 but not the other accessions (*p* < 0.05).

Table S2 shows the variations in crude protein, total fat, crude fiber, and dietary fiber contents among the yardlong bean accessions with different pod and seed colors. The ANOVA results are summarized in Table S3. The results showed that pod color (P), seed color (S), and P × S interaction had no significant effects on any of the nutritional parameters. Dietary fiber content was the only exception, as it was significantly affected by P × S interaction (*p* < 0.01). Specifically, accessions with green pods and black seeds exhibited the highest average dietary fiber content (21.68%), while accessions with light-green pods with black seeds had the lowest average dietary fiber content (16.12%).

#### 3.1.2. Vitamin C Content

**Figure 2:**
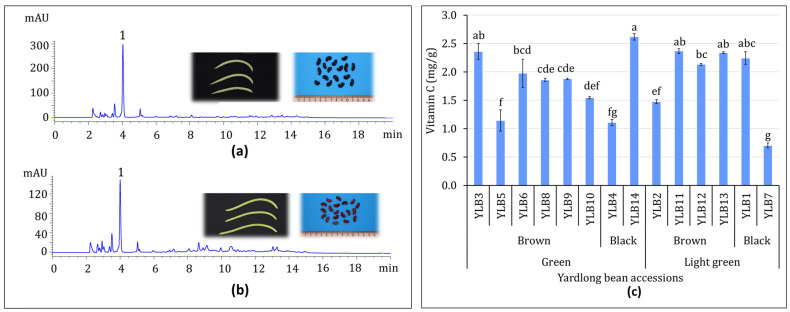
LC-chromatogram of vitamin C in representative samples with light green pod and black seed color (**a**) and green pod and brown seed color (**b**), and the content of vitamin C in 14 yardlong bean accessions (**c**). Peak assignment: 1: Vitamin C. Different letters on bars indicate significantly different mean values at *p* < 0.05.

The vitamin C level in each of the 14 yardlong bean accessions was examined using HPLC, as described before (Figure 2a,b). Similar to other nutrients, the levels of vitamin C showed a wide variation, with a variance of 29.82%. This variance was higher than any of the nutritional components described before, except for total fat content. With a mean of 1.84 mg/g, the vitamin C content ranged from 0.70 to 2.62 mg/g (Figure 2c). Accession YLB14 was found to have the highest vitamin C content (Figure 2c). This accession had the second highest DFC (21.45%) and the third highest total fat content (1.44%). Conversely, accession YLB7, which had the lowest total fat content, exhibited the lowest vitamin C content. The vitamin C content in YLB14 differed significantly from all other accessions, except for YLB1, YLB3, YLB11, and YLB13 (*p* < 0.05). The effects of pod color and seed color variations on vitamin C level were similarly analyzed (Tables S2 and S3). Although accessions with light-green pods and brown seeds displayed higher average vitamin C contents, pod color and seed color differences as well as P × S interaction, once again, did not show significant effects.

#### 3.1.3. Individual and Total Fatty Acid Contents

The contents of palmitic acid, stearic acid, oleic acid, linoleic acid, and linolenic acid in each of the 14 yardlong bean accessions are displayed in Table 1, and all showed significant variations (*p* < 0.05). Likewise, the levels of omega-6 (ω6) to omega-3 (ω3) ratio, IA, and IT were in the ranges of 1.75–2.67, 0.43–0.49, and 0.38–0.57, respectively (Table 1). Accession YLB8 had the highest palmitic acid content (31.36%) and TSFA (35.58%), with the former being significantly different from the other accessions. The TSFA in accession YLB8 was also significantly different from all accessions except YLB4 (35.37%). Similarly, accession YLB11 contained the highest stearic acid (4.65%), which was significantly different from the other accessions. The lowest palmitic acid and stearic acid contents were found in accessions YLB3 (28.87%) and YLB10 (3.17%), respectively. Similarly, the highest OA content found in accession YLB13 (15.87%) was significantly different from all other accessions except for YLB2 (14.90%) and YLB10 (14.39%). Conversely, the highest content of linoleic acid in accession YLB9 (41.06%) was significantly different from all other accessions except for accessions YLB14 (40.39%) and YLB4 (39.83%). The highest linolenic acid content found in accession YLB12 (19.69%) was significantly different from all other accessions (*p* < 0.05). Across all yardlong bean accessions, the level of TUFA was higher than TSFA, with PUFA contributing to more than 76% of the TUFA (Figure S1).

The variations in individual and total fatty acid contents among yardlong beans of different pod and seed colors were also statistically analyzed (Tables S2 and S3). Except for oleic acid and linoleic acid, pod and seed color variations as well as P × S interaction had no significant effect on any of the other fatty acids, including total contents. Compared to accessions with light-green pods, those with green pods exhibited higher average oleic acid and linoleic acid contents. Likewise, black seeded accessions had a higher average linoleic acid content and a lower average oleic acid content compared to brown seeded accessions. Overall, accessions with light-green pods with brown seeds showed the highest average oleic acid content (14.06%) as well as the lowest average linoleic acid content (35.84%). On the other hand, accessions with green pods and black seeds exhibited the highest average linoleic acid (40.11%) and the lowest average oleic acid (9.80%) contents. As shown in Table S2, the differences in oleic acid and linoleic acid between the highest and lowest average values were statistically significant (*p* < 0.05).

### 3.2. Total Metabolite Contents

The total secondary metabolite contents in each of the yardlong bean accessions are provided in Table 2. All the contents, including TPC, TTC, and TSC, showed significant variations among the yardlong beans (*p* < 0.05) and showed variances of 18.74, 27.56, and 20.98%, respectively. Accession YLB4 exhibited the highest levels of TPC (7.86 mg GAE/g), TTC (90.76 mg CE/g), and TSC (62.52 mg DE/g), with the first two being significantly different from the other accessions (*p* < 0.05). The highest TSC in YLB4 was also significantly different from the other accessions, except for YLB7, YLB11, and YLB9, which had the second (61.80 mg DE/g), third (56.58 mg DE/g), and fourth (54.98 mg DE/g) highest TSC. Conversely, accession YLB13 had the lowest TPC (3.99 mg GAE/g), YLB12 the lowest TTC (36.94 mg CE/g), and YLB6 the lowest TSC (31.80 mg DE/g).

Compared to the nutritional components previously described, the statistical analysis showed that seed color variation significantly affected TPC and TTC (Tables S2 and S3). On the other hand, pod color variation only had a significant impact on TPC. Specifically, accessions with black seeds had higher average TPC (6.15 mg GAE/g), TTC (65.46 mg CE/g), and TSC (53.50 mg/g) compared to those with brown seeds (Table S2). Similarly, accessions with green pods had higher average TPC (6.11 mg GAE/g) but lower TTC (55.00 mg CE/g) and TSC (48.06 mg DE/g) levels than those with light-green pods. Statistical analysis of the interaction between pod and seed color revealed that accessions with green pods and black seeds had the highest average TPC (7.43 mg GAE/g), TTC (67.83 mg CE/g), and TSC (57.18). On the other hand, accessions with light-green pods and brown seeds showed the lowest average TPC (4.69 mg GAE/g), while those with green pods and brown seeds had the lowest average TTC (43.87 mg CE/g) and TSC (41.75 mg DE/g) levels. The differences in TPC and TTC between the highest and lowest average values were found to be statistically significant (*p* < 0.05). However, only the TPC level was significantly affected by the interaction between pod color and seed color (Table S3).

### 3.3. Antioxidant Activities

The antioxidant properties of the 14 yardlong bean accessions were evaluated using three separate assays (Table 2). All three assays, including DPPH^•^ scavenging activity, ABTS^•+^ scavenging activity, and RP, exhibited more than 20.00% variance. Among the accessions, YLB4 exhibited the maximum antioxidant index, having the highest levels of DPPH^•^ scavenging activity (9.51 mg AAE/g), ABTS^•+^ scavenging activity (16.76 mg TE/g), and RP (9.48 mg AAE/g), all being significantly higher than the other accessions (*p* < 0.05). In contrast, YLB5 exhibited the lowest antioxidant index and had the lowest ABTS^•+^ scavenging activity (6.05 mg AAE/g) and RP (2.70 mg AAE/g) levels, while YLB12 had the lowest DPPH^•^ scavenging activity (1.86 mg AAE/g).

In terms of pod color, accessions with green pods had higher average antioxidant activities compared to light-green pods (Tables S2 and S3). Similarly, black seeded yardlong beans showed significantly higher antioxidant activities compared to accessions with brown seeds (*p* < 0.05). A comparative analysis among green pod and light-green pod yardlong beans of different seed colors showed that the average DPPH^•^ scavenging activity decreased in the order of green pods with black seeds (6.92 mg AAE/g) > light-green pods with black seeds (4.00 mg AAE/g) > green pods with brown seeds (3.11 mg AAE/g) > light-green pods with brown seeds (2.41 mg AAE/g). Likewise, the average ABTS^•+^ scavenging activity and RP decreased in the order of green pods with black seeds (14.02 mg TE/g and 8.20 mg AAE/g, respectively) > light-green pods with black seeds (9.26 mg TE/g and 5.65 mg AAE/g, respectively) > light-green pods with brown seeds (7.41 mg TE/g and 4.05 mg AAE/g, respectively) > green pods with brown seeds (7.12 mg TE/g and 3.69 mg AAE/g, respectively). Each of these variations indicated significant differences (Table S3).

### 3.4. Cluster, Principal Components, and Correlation Analyses

**Figure 3:**
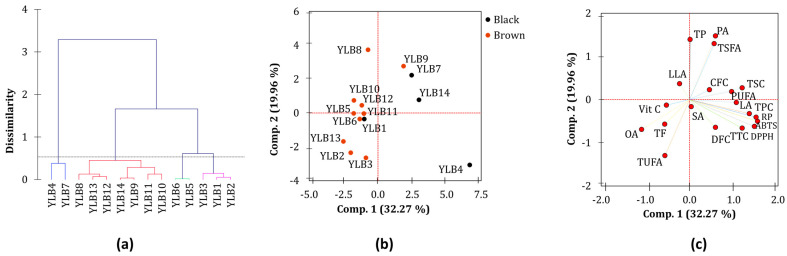
Dendrogram of 14 yardlong bean accessions (**a**), score plot of yardlong bean accessions according to seed color (**b**) and loading plot of variable (**c**) from PCA. ABTS: ABTS^•+^ scavenging activity, CFC: crude fiber content, DFC: dietary fiber content, DPPH: DPPH^•^ scavenging activity, LA: linoleic acid, LLA: linolenic acid, OA: oleic acid, PA: palmitic acid, PUAF: total polyunsaturated fatty acid, RP: reducing power, SA: stearic acid, TF: total fat, TP: total protein, TSFA: total saturated fatty acid, TUFA: total unsaturated fatty acid, TPC: total phenolic content, TSC: total saponin content, TTC: total tannin content, Vit C: vitamin C.

Cluster analysis and principal component analysis (PCA) were conducted using the whole dataset to examine how the yardlong bean accessions relate to their nutritional components, metabolite contents, and antioxidant activities. The cluster analysis grouped the yardlong bean accessions into four categories (Figure 3a). The first and third groups each consisted of two accessions, while the second group had seven accessions, and the fourth group had three accessions. The first two components in the PCA, among those with Eigenvalues greater than 1, explained 52.23% of the total variance. Score plot analysis was carried out using seed color (Figure 3b) and pod color (Figure S2) as explanatory variables across these components. The loading plot showed a wide spread of the examined parameters on the two principal components (Figure 3c) and demonstrated different contributions to the observed variance (Table S2) and degrees of association between them.

**Figure 4:**
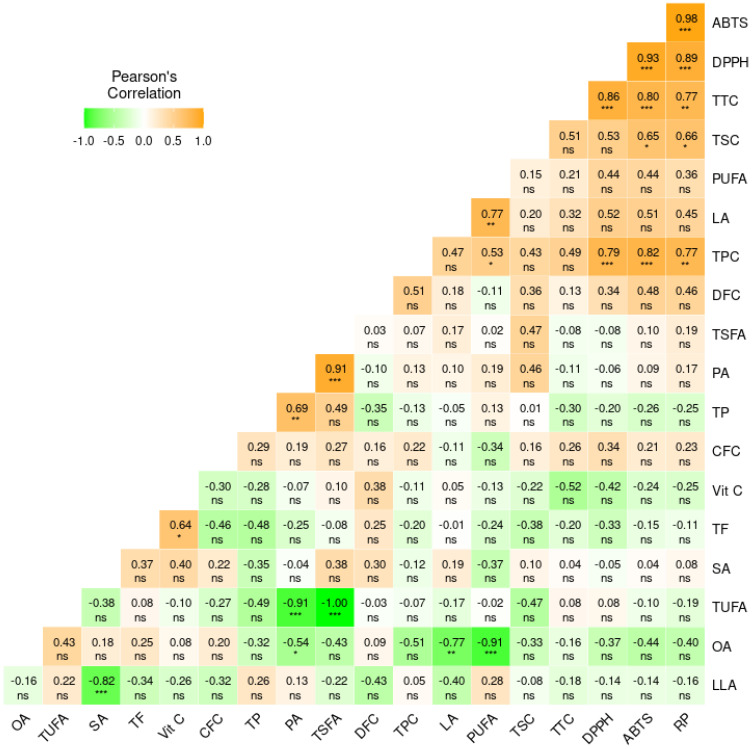
Pearson’s correlation matrix showing the association between the analyzed quantitative parameters. *** *p* < 0.001, ** *p* < 0.01, * *p* < 0.05, ns: not significant. ABTS: ABTS^•+^ scavenging activity, CFC: crude fiber content, DFC: dietary fiber content, DPPH: DPPH^•^ scavenging activity, LA: linoleic acid, LLA: linolenic acid, OA: oleic acid, PA: palmitic acid, PUFA: total polyunsaturated fatty acid, RP: reducing power, SA: stearic acid, TF: total fat, TP: total protein, TSFA: total saturated fatty acid, TUFA: total unsaturated fatty acid, TPC: total phenolic content, TSC: total saponin content, TTC: total tannin content, Vit C: vitamin C.

Pearson’s correlation analysis showed diverse relationships between the parameters examined at different levels of significance (Figure 4). TPC, TSC, and TTC displayed strong and positive correlations with all antioxidant activities (r ≥ 0.53) and with each other (r ≥ 0.51) at different levels of significance. TPC had a positive and moderate correlation with dietary fiber content (DFC) (r = 0.51), although the correlation was not significant. Likewise, DFC and total fat had negative correlations with crude protein content (r = −0.35 and −0.48, respectively), while vitamin C showed a moderate and positive correlation with total fat (r = 0.64, *p* < 0.05). Unsaturated fatty acids showed negative correlations with each other, with oleic acid and linoleic acid showing a strong and significant correlation (r = −0.77, *p* < 0.01). In contrast to other fatty acids, linoleic acid also displayed positive correlations with TPC (r = 0.47), TTC (r = 0.32), TSC (r = 0.20), and antioxidant activities (r ≥ 0.45). Palmitic acid and linoleic acid showed strong and significant correlations with TSFA (r = 0.91, *p* < 0.001) and PUFA (r = 0.77, *p* < 0.01), respectively.

## 4. Discussion

### 4.1. Variations in Biochemical Components

Yardlong beans have numerous health benefits for humans, but they remain one of the underutilized legumes [31]. On the other hand, the biochemical compositions of plants, in general, are influenced by genetic variances, environmental factors, and pre- and post-harvest practices [13]. Understanding how these factors affect the chemical components is crucial for selecting plant materials with desirable qualities for the food industries and breeding programs [13,32]. This study revealed significant differences in all the nutritional components, secondary metabolite contents, and antioxidant activities among the 14 field-grown yardlong bean accessions. The variances observed in the contents of nutritional components, such as total fat, crude fiber, dietary fiber, vitamin C, and fatty acids, except for palmitic acid and linoleic acid, exceeded 8.00%. Furthermore, there was more than 18.00% variances in total secondary metabolite contents and over 31.00% variances in antioxidant activities. These findings suggest a wide genetic diversity among the investigated yardlong bean accessions [3].

Previous studies have also analyzed the various biochemical components of yardlong beans, and the reported values were wide-ranging. A recent study in Bangladesh examined matured pods of five yardlong bean varieties and reported crude protein levels ranging from 2.80 to 3.30 g/100 g, total fat levels between 0.10 and 0.19 g/100 g, crude fiber levels of 1.23–1.85 g/100 g, and vitamin C levels between 18.20 and 20.22 mg/100 g [5]. All of these values were much lower than those observed in this study. The total protein content found in this study fell within the range found in six cowpea cultivars grown in Nigeria (20.5–39.7%) [6]. The same study reported a much lower crude fiber content (1.70–4.50%). Another study conducted on 24 Chinese cowpea cultivars found a similar protein content (17.30–27.23%) but a higher fat content (1.87–3.14%) compared to the findings of this study [7]. The differences observed in the reported values could be attributed to variations in growing conditions, analysis methods, plant parts examined, and genetic factors, among others [13]. The analysis of fatty acids in this study also identified palmitic acid and linoleic acid as the main saturated and unsaturated fatty acids, respectively, which was consistent with previous findings [3]. The lipid quality of the yardlong bean accessions was evaluated based on the ω6:ω3 ratio, IA, and IT indices. All were estimated from the analyzed fatty acid and ranged between 1.75 and 2.67 (ω6:ω3 ratio), 0.43–0.49 (IA), and 0.38–0.57 (IT). It was recommended that maintaining an optimal balance between omega-6 and omega-3 fatty acids in both foods and diet could help reduce the risk of chronic illnesses. Specifically, a ω6:ω3 ratio between 1.5 and 3.0 is considered adequate [33]. Interestingly, all 14 yardlong bean accessions had a ω6:ω3 ratio within the desired range. On the other hand, IA and IT indices are important indicators of lipid quality in foods and food products. While there have been no established reference values for yardlong bean oils, the IA and IT values of the 14 accessions were comparable or close to those of commonly used oils such as olive oil, corn oil, and avocado oil [28]. These findings suggest that the yardlong bean accessions could serve as valuable sources of healthy lipids [28,33].

The yardlong bean accessions also showed wide variations in terms of their secondary metabolite contents and antioxidant activity levels, as described before. Compared to the nutritional factors discussed earlier, there was limited information on the total secondary metabolite levels and antioxidant properties of yardlong beans. The values reported in the existing literature for related species also differ in measurement units, analysis protocols, and plant parts examined, making comparison difficult [34,35,36]. Overall, in this study, yardlong bean accessions with higher levels of phenolic contents and antioxidant activities demonstrated an increased antioxidant index. For instance, accession YLB4, which showed the highest TPC, TTC, and TSC, also exhibited the highest antioxidant activities, including DPPH^•^ scavenging activity, ABTS^•+^ scavenging activity, and RP. Additionally, this specific accession had the highest DFC. On the other hand, accession YLB14 displayed the second highest levels of TPC, ABTS^•+^ scavenging activity, and RP, and the third highest level of DPPH^•^ scavenging activity. Overall, the results observed once again highlight the role of polyphenols in yardlong beans as antioxidants [9,30,37]. Therefore, future studies should focus on identifying specific phenolic compounds responsible for their antioxidant activities [38].

### 4.2. Effects of Pod Color and Seed Color Differences

This study also examined how differences in pod color and seed color influence the biochemical components analyzed for the first time. The findings on nutritional components indicated that pod color, seed color, or their interactions did not have a significant effect on the levels of crude protein, total fat, crude fiber, and vitamin C. However, the dietary fiber content was significantly affected by the interaction of pod color and seed color. This could be due to the significantly high levels of dietary fiber content found in yardlong beans with green pods and black seeds. Similar to the nutritional properties stated before, the variations in pod and seed colors did not have significant effects on the levels of fatty acids analyzed. Despite the lack of prior research on yardlong beans, comparable studies on other legumes, such as soybeans, have shown different results. Two separate studies by Cho et al. [39] and Lee et al. [40] did not find any significant variations in total protein and total fat levels among soybeans of different seed colors, which supports our findings [39,40]. The former study also found that seed color variation did not have a significant effect on the fatty acid contents, while the latter noted a significant difference in linolenic acid content only. Our latest research found significant differences in protein and fat levels among black and yellow soybeans, as well as a significantly low level of palmitic acid in green soybeans compared to other colors [41]. Overall, the results of this study suggest that the color of yardlong bean seeds or immature pods may not accurately indicate their nutritional contents. Thus, consuming yardlong beans with different pod and seed colors may not lead to significant variations in nutrient intake. Nonetheless, additional research on the nutritional content of other types of colored pods and seeds is recommended before reaching a concluding remark. In contrast to the nutritional components, variations in seed color and pod color, as well as their interactions, had significant effects on the total secondary metabolite contents and antioxidant activities, including TPC, DPPH^•^ scavenging activity, ABTS^•+^ scavenging activity, and RP. TTC was significantly influenced only by seed color variation, while TSC remained unaffected by any of the factors. These findings were consistent with prior studies that have reported significant differences in total metabolite levels and antioxidant properties among legumes with varying seed colors [39,40,41,42]. In this study, yardlong beans with green immature pods and black seeds were identified as promising sources of antioxidants owing to their high phenolic content and increased antioxidant activities. Previous studies have also highlighted that legumes with black seeds, even those from different genera, display similar properties [43,44,45]. In summary, this study signified that pod and seed colors may serve as indicators of antioxidant levels in yardlong beans. However, further investigation involving pods and seeds of different colors is required once again to validate these observations [15,26].

### 4.3. Multivariate Analysis

The entire biochemical dataset was analyzed using cluster analysis, PCA, and correlation analysis to further view the connection between the yardlong bean accessions and these parameters. The cluster analysis and PCA did not distinctly categorize the yardlong bean accessions based on their pod or seed color. Moreover, most of the parameters examined did not exhibit significant variations among the groups in the cluster analysis, with the exception of total fat, antioxidant activities, TTC, and TSC. Nonetheless, both accessions in cluster one were black seed accessions and had significantly high levels of antioxidant activities, TTC, and TSC, along with a low total fat content. The second group primarily consisted of accessions with brown seeds, four of which had green pods. The third group contained accessions with green pods and brown seeds. In the PCA, the yardlong beans with black seeds tended to be grouped on the positive side of component 1. Most of the brown yardlong beans, except for YLB9, were clustered on the negative side of component 1. The main factors contributing to the variance observed along component 1 were TPC, TTC, DPPH^•^ scavenging activity, ABTS^•+^ scavenging activity, and RP, contributing between 8.41 and 14.08% (Table S3). It is worth noting that these parameters were significantly affected by pod and/or seed color variations. On the other hand, crude protein (18.70%), palmitic acid (20.91%), TSFA (16.19%), and TUFA (16.20%) were the major contributors to the variance observed along component 2. Pearson’s correlation analysis also supported the findings of the PCA loading plot and demonstrated wide-ranging associations between the parameters analyzed. For instance, the strong and significant correlation between total secondary metabolite contents and antioxidant activities highlights the importance of yardlong bean phenolic compounds in neutralizing harmful radicals [46]. The negative and significant correlation between oleic acid and linoleic acid observed in this study also aligns with previous findings in other legumes. Such associations between these unsaturated fatty acids may be attributed to the effect of enzymes that regulate their interconversion [33,39,40]. The positive correlations of linoleic acid with total secondary metabolite contents as well as antioxidant activities suggest its potential for combating reactive free radicals [32,47].

## 5. Conclusions

This study investigated the contents of major nutritional components, secondary metabolites, and antioxidant activities of 14 yardlong bean accessions grown in an experimental field while also examining the effects pod and seed color variations. The results showed significant differences in all the analyzed biochemical components among the yardlong bean accessions. In general, those accessions with superior qualities could be advantageous for the food industry. Statistical analysis among yardlong beans of different pod and seed colors indicated that those with green pods and black seeds had significantly high levels of dietary fiber, linoleic acid, total tannin, total phenol, DPPH^•^ scavenging activity, and ABTS^•+^ scavenging activity. Despite the small sample size, principal component analysis emphasized the importance of antioxidant parameters in the observed variations, revealing strong correlations among them. Therefore, pod and seed colors could be used as distinguishing parameters for different yardlong bean varieties based on their overall antioxidant index. In contrast, pod and seed colors might not be crucial factors in assessing the nutritional qualities of yardlong beans. Further investigations, considering large sample sizes of yardlong beans with various pod and seed colors, are suggested to verify these observations.

## Figures and Tables

**Table 1:** Contents of individual fatty acids across 14 yardlong bean accessions cultivated in Korea.

Pod Color	Seed Color	Accessions	Palmitic Acid (%)	Stearic Acid (%)	Oleic Acid (%)	Linoleic Acid (%)	Linolenic Acid (%)	ω6:ω3	IA	IT
**Green**	Brown	YLB3	28.87 ± 0.01 ^i^	3.91 ± 0.02 ^de^	13.65 ± 0.04 ^bc^	37.27 ± 0.05 ^bc^	16.30 ± 0.05 ^e^	2.29	0.43	0.44
		YLB5	29.24 ± 0.60 ^g^	3.69 ± 0.00 ^f^	11.47 ± 0.07 ^de^	37.95 ± 0.05 ^b^	17.66 ± 0.06 ^cd^	2.15	0.44	0.47
		YLB6	29.25 ± 0.60 ^g^	3.75 ± 0.02 ^def^	11.89 ± 0.01 ^de^	37.20 ± 0.10 ^bc^	17.92 ± 0.04 ^c^	2.08	0.44	0.48
		YLB8	31.36 ± 0.02 ^a^	4.22 ± 0.02 ^c^	12.74 ± 0.07 ^cd^	36.05 ± 0.07 ^cde^	15.63 ± 0.04 ^f^	2.30	0.49	0.43
		YLB9	31.07 ± 0.11 ^b^	3.56 ± 0.02 ^ef^	7.26 ± 0.06 ^g^	41.06 ± 0.08 ^a^	17.05 ± 0.01 ^e^	2.39	0.48	0.42
		YLB10	30.30 ± 0.05 ^d^	3.17 ± 0.03 ^g^	14.39 ± 0.03 ^abc^	33.28 ± 0.06 ^f^	18.87 ± 0.08 ^b^	1.76	0.46	0.57
	Black	YLB4	29.52 ± 0.70 ^f^	3.83 ± 0.02 ^def^	10.50 ± 0.02 ^ef^	39.83 ± 0.05 ^a^	16.32 ± 0.04 ^e^	2.44	0.44	0.41
		YLB14	30.93 ± 0.08 ^c^	4.44 ± 0.01 ^b^	9.09 ± 0.05 ^fg^	40.39 ± 0.06 ^a^	15.15 ± 0.05 ^fg^	2.67	0.48	0.38
**Light green**	Brown	YLB2	28.99 ± 0.05 ^hi^	4.20 ± 0.02 ^c^	14.90 ± 0.08 ^ab^	36.97 ± 0.04 ^bc^	14.93 ± 0.06 ^g^	2.48	0.43	0.40
		YLB11	29.78 ± 0.03 ^e^	4.65 ± 0.01 ^a^	13.59 ± 0.05 ^bc^	36.54 ± 0.03 ^bcd^	15.44 ± 0.05 ^f^	2.37	0.45	0.42
		YLB12	30.31 ± 0.03 ^d^	3.63 ± 0.02 ^f^	11.89 ± 0.07 ^de^	34.48 ± 0.05 ^ef^	19.69 ± 0.10 ^a^	1.75	0.46	0.57
		YLB13	29.09 ± 0.03 ^h^	4.26 ± 0.01 ^bc^	15.87 ± 0.08 ^a^	35.35 ± 0.07 ^de^	15.44 ± 0.03 ^f^	2.29	0.44	0.44
	Black	YLB1	29.87 ± 0.12 ^e^	3.91 ± 0.01 ^de^	11.40 ± 0.26 ^de^	37.53 ± 0.17 ^bc^	17.28 ± 0.09 ^d^	2.17	0.45	0.46
		YLB7	31.05 ± 0.50 ^bc^	3.94 ± 0.02 ^d^	11.37 ± 0.03 ^de^	36.11 ± 0.09 ^cd^	17.52 ± 0.07 ^cd^	2.06	0.48	0.49
	Total	Min	28.87	3.17	7.26	33.28	14.93	1.75	0.43	0.38
		Max	31.36	4.65	15.87	41.06	19.69	2.67	0.49	0.57
		Mean	29.97	3.94	12.14	37.14	16.80	2.23	0.46	0.46
		CV (%)	2.78	9.44	18.26	5.66	8.30	10.81	4.15	11.71

Different superscript letters in a column indicate significantly different mean values (*p* < 0.05). IA: index of atherogenicity; IT: Index of thrombogenicity.

**Table 2:** Total metabolite contents and antioxidant activities of 14 yardlong bean accessions cultivated in Korea.

Pod Color	Seed Color	Accession	Total Phenol (mg GAE/g)	Total Tannin (mg CE/g)	Total Saponin (mg DE/g)	DPPH (mg AAE/g)	ABTS (mg TE/g)	RP (mg AAE/g)	AI
**Green**	Brown	YLB3	5.83 ± 0.26 ^c^	49.65 ± 1.42 ^de^	39.84 ± 1.90 ^def^	3.94 ± 0.38 ^cd^	7.96 ± 0.80 ^c^	4.38 ± 0.42 ^cd^	54.61
		YLB5	4.84 ± 0.32 ^def^	40.97 ± 2. 19 ^fg^	34.38 ± 1.03 ^f^	2.82 ± 0.37 ^efg^	6.05 ± 0.63 ^d^	2.70 ± 0.44 ^f^	42.65
		YLB6	5.64 ± 0.37 ^cd^	42.36 ± 1.71 ^efg^	31.80 ± 1.72 ^f^	3.51 ± 0.21 ^de^	7.02 ± 0.66 ^cd^	3.47 ± 0.27 ^ef^	47.47
		YLB8	4.84 ± 0.09 ^def^	39.69 ± 1.41 ^fg^	40.83 ± 1.53 ^def^	2.26 ± 0.09 ^gh^	6.37 ± 0.13 ^d^	3.46 ± 0.17 ^ef^	44.82
		YLB9	5.18 ± 0.24 ^c–f^	44.66 ± 1. 86 ^d–g^	54.98 ± 0.64 ^abc^	3.42 ± 0.16 ^de^	8.39 ± 0.38 ^c^	4.45 ± 0.40 ^cd^	56.02
		YLB10	5.23 ± 0.42 ^c–f^	45.90 ± 3.39 ^def^	48.67 ± 6.80 ^bcd^	2.73 ± 0.31 ^efg^	6.93 ± 0.63 ^cd^	3.70 ± 0.32 ^de^	5068
	Black	YLB4	7.86 ± 0.19 ^a^	90.76 ± 2.08 ^a^	62.52 ± 3.55 ^a^	9.51 ± 0.45 ^a^	16.76 ± 0.63 ^a^	9.48 ± 0.28 ^a^	100.00
		YLB14	7.00 ± 0.41 ^b^	44.90 ± 3.49 ^d–g^	51.84 ± 9.11 ^bc^	4.32 ± 0.75 ^bc^	11.29 ± 1.15 ^b^	6.92 ± 0.45 ^b^	67.88
**Light green**	Brown	YLB2	4.42 ± 0.52 ^fg^	58.59 ± 7.08 ^c^	35.73 ± 2.26 ^f^	2.85 ± 0.44 ^efg^	7.09 ± 0.62 ^cd^	4.14 ± 0.42 ^cde^	48.98
		YLB11	4.64 ± 0.34 ^efg^	52.11 ± 2.69 ^cd^	56.58 ± 7.59 ^ab^	2.48 ± 0.19 ^fgh^	7.17 ± 0.31 ^cd^	3.33 ± 0.35 ^eg^	51.83
		YLB12	5.69 ± 0.46 ^c^	36.94 ± 6.58 ^g^	46.25 ± 1.02 ^cde^	1.86 ± 0.32 ^h^	8.00 ± 1.06 ^c^	4.44 ± 0.33 ^cd^	5020
		YLB13	3.99 ± 0.54 ^g^	38.07 ± 2.64 ^fg^	46.72 ± 3.94 ^cde^	2.45 ± 0.27 ^fgh^	7.38 ± 0.84 ^cd^	4.28 ± 0.44 ^cd^	47.08
	Black	YLB1	4.43 ± 0.20 ^fg^	58.82 ± 2.03 ^c^	37.83 ± 0.80 ^ef^	3.14 ± 0.27 ^ef^	8.47 ± 0.60 ^c^	4.75 ± 0.29 ^c^	52.56
		YLB7	5.29 ± 0.30 ^cde^	67.38 ± 4.77 ^b^	61.80 ± 0.43 ^a^	4.86 ± 0.19 ^b^	10.05 ± 0.28 ^b^	6.55 ± 0.28 ^b^	70.10
	Total	Min	3.99	36.94	31.80	1.86	6.05	2.70	42.65
		Max	7.86	90.76	62.52	9.51	16.76	9.48	100.00
		Mean	5.35	50.77	46.41	3.58	8.50	4.72	56.06
		CV (%)	18.74	27.56	20.98	51.01	31.32	36.62	25.56

Different superscript letters in a column indicate significantly different mean values (*p* < 0.05). ABTS: ABTS^•+^ scavenging activity, DPPH: DPPH^•^ scavenging activity, RP: reducing power.

## Data Availability

All the data related to this study are incorporated in the manuscript and Supplementary Materials. Further inquiries can be directed to the first author.

## Supplementary Materials

The following supporting information can be downloaded at: https://www.mdpi.com/article/10.3390/antiox13091134/s1, Figure S1: The contents of total fatty acids across 14 yardlong bean accessions. Figure S2: Bi-plot of yardlong bean accessions according to pod color and analyzed variables along the first and second principal components. Table S1: General information of the 14 yardlong bean accessions investigated; Table S2: Analysis of variance data showing the variations of nutritional components, total secondary metabolites, and antioxidant activities across yardlong beans of different pod and seed colors; Table S3: Analysis of variance data on the effects of pod color and seed color variations on the analyzed parameters in yardlong beans and contributions of the analyzed parameters to the variance observed in the principal component analysis (PCA).
