# Supplementary material for: Nutritional Qualities, Metabolite Contents, and Antioxidant Capacities of Yardlong Beans (Vigna unguiculata subsp. sesquipedalis) of Different Pod and Seed Colors
Source: Antioxidants (Basel). 2024 Sep 19;13(9):1134. doi: 10.3390/antiox13091134 (PMC11428976; doi:10.3390/antiox13091134)
Supplement: Supplementary file 1 [file antioxidants-13-01134-s001.zip › antioxidants-3196787-supplementary.pdf]

Supplementary material

Nutritional Qualities, Metabolite Contents, and Antioxidant Capacities of Yardlong Beans (*Vigna unguiculata* subsp. *sesquipedalis)* of Different Pod and Seed Colors

Yu-Mi Choi [1], Myoung-Jae Shin [1], Hyemyeong Yoon [1], Sukyeung Lee [2], Jungyoon Yi [1], Xiaohan Wang [3], and Kebede Taye Desta [1,*]

[1] National Agrobiodiversity Center, National Institute of Agricultural Sciences, Rural Development Administration, Jeonju 54874, Republic of Korea.
[2] International Technology Cooperation Center, Technology Cooperation Bureau, Rural Development Administration, Jeonju 54875, Republic of Korea.
[3] Institute of Crop Sciences, Chinese Academy of Agricultural Sciences, Beijing 100081, China
* Correspondence: kebedetdesta@korea.kr

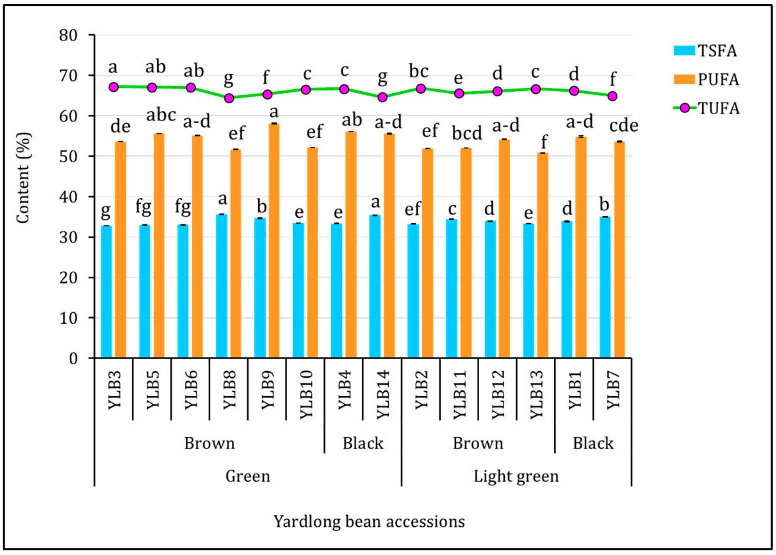

Figure S1. The contents of total fatty acids across 14 yardlong bean accessions. Different letters on bars (charts) indicate significantly different means at $p < 0.05$. PUFA: polyunsaturated fatty acid, TSFA: Total saturated fatty acid, TUFA: total unsaturated fatty acid.

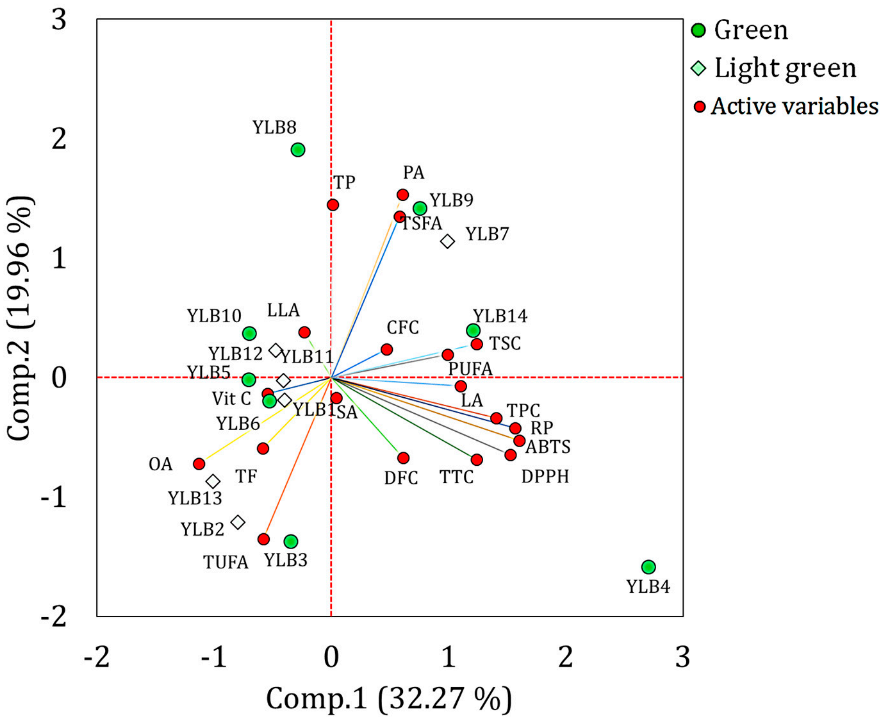

Figure S2. Bi-plot of yardlong bean accessions according to pod color and analyzed variables along the first and second principal components. ABTS: ABTS•+ scavenging activity, CFC: crude fiber content, DFC: dietary fiber content, DPPH: DPPH• scavenging activity, LA: linoleic acid, LLA: linolenic acid, OA: oleic acid, PA: palmitic acid, PUAF: total polyunsaturated fatty acid, RP: reducing power, SA: stearic acid, TF: total fat, TP: total protein, TSFA: total saturated fatty acid, TUFA: total unsaturated fatty acid, TPC: total phenolic content, TSC: total saponin content, TTC: total tannin content, Vit C: vitamin C.

Table S1. General information of the 14 yardlong bean accessions investigated.

Introduction (IT)/ Temporary (K) number	Code	Origin	Immature pod color	Seed color
IT160312	YLB1	China	Light green	Black
IT160314	YLB2	China	Light green	Brown
IT181267	YLB3	Philippines	Green	Brown
IT181271	YLB4	Philippines	Green	Black
IT181272	YLB5	Philippines	Green	Brown
IT181273	YLB6	Philippines	Green	Brown
IT189634	YLB7	Taiwan	Light green	Black
IT212205	YLB8	Thailand	Green	Brown
IT212207	YLB9	Thailand	Green	Brown
IT212208	YLB10	Thailand	Green	Brown
K003735	YLB11	China	Light green	Brown
K153675	YLB12	Pakistan	Light green	Brown
K166120	YLB13	China	Light green	Brown
K243956	YLB14	Malaysia	Green	Black

Table S2. Analysis of variance data showing the variations of nutritional components, total secondary metabolites, and antioxidant activities across yardlong beans of different pod and seed colors.

Parameter	Pod color	Values	Seed color		Total
			Brown	Black	
Crude protein (%)	Light green	*n*	4	2	6
		Min.	25.15	25.66	25.15
		Max.	25.57	27.10	27.10
		Mean	$25.36^{A,a}$	$26.38^{A,a}$	25.70^{a}
	Green	*n*	6	2	8
		Min.	24.31	25.33	24.31
		Max.	29.10	26.02	29.10
		Mean	$27.24^{A,a}$	$25.68^{A,a}$	26.85^{a}
		Total mean	26.49^{A}	26.03^{A}	26.36
Total fat (%)	Light green	*n*	4	2	6
		Min.	0.96	0.33	0.33
		Max.	1.49	1.45	1.49
		Mean	$1.15^{A,a}$	$0.89^{A,a}$	1.06^{a}
	Green	*n*	6	2	8
		Min.	0.65	0.60	0.60
		Max.	0.95	1.44	1.44
		Mean	$0.77^{A,a}$	$1.02^{A,a}$	0.83^{a}
		Total mean	0.92^{A}	0.96^{A}	0.93
Crude fiber (%)	Light green	*n*	4	2	6
		Min.	4.37	4.37	4.37
		Max.	4.92	5.35	5.35
		Mean	$4.70^{A,a}$	$4.86^{A,a}$	4.75^{a}
	Green	*n*	6	2	8
		Min.	4.35	4.76	4.35
		Max.	6.14	5.50	6.14
		Mean	$5.03^{A,a}$	$5.13^{A,a}$	5.06^{a}
		Total mean	4.90^{A}	5.00^{A}	4.92
Dietary fiber (%)	Light green	*n*	4	2	6
		Min.	19.37	15.92	15.92
		Max.	20.90	16.31	20.90
		Mean	$19.86^{A,a}$	$16.11^{B,b}$	18.61^{a}
	Green	*n*	6	2	8
		Min.	15.83	21.45	15.83
		Max.	19.87	21.90	21.90
		Mean	$18.33^{B,b}$	$21.68^{A,a}$	19.17^{a}
		Total mean	18.94^{A}	18.89^{A}	19.83
Vitamin C (mg/g)	Light green	*n*	4	2	6
		Min.	1.48	0.70	0.70
		Max.	2.37	2.24	2.37
		Mean	$2.08^{A,a}$	$1.47^{A,a}$	1.88^{a}
	Green	*n*	6	2	8
		Min.	1.14	1.11	1.11
		Max.	2.36	2.62	2.62
		Mean	$1.79^{A,a}$	$1.86^{A,a}$	1.81^{a}

		Total mean	1.91^{A}	1.67^{A}	1.84
Palmitic acid (%)	Light green	*n*	4	2	6
		Min.	28.99	29.87	28.99
		Max.	30.31	31.05	31.05
		Mean	29.54^{A,a}	30.46^{A,a}	29.85^{a}
	Green	*n*	6	2	8
		Min.	28.87	29.52	28.87
		Max.	31.36	30.93	31.36
		Mean	30.01^{A,a}	30.23^{A,a}	30.07^{a}
		Total mean	29.83^{A}	30.34^{A}	29.97
Stearic acid (%)	Light green	*n*	4	2	6
		Min.	3.63	3.91	3.63
		Max.	4.65	3.94	4.65
		Mean	4.18^{A,a}	3.93^{A,a}	4.10^{a}
	Green	*n*	6	2	8
		Min.	3.17	3.83	3.17
		Max.	4.22	4.44	4.44
		Mean	3.72^{A,a}	4.13^{A,a}	3.82^{a}
		Total mean	3.90^{A}	4.03^{A}	3.94
Oleic acid (%)	Light green	*n*	4	2	6
		Min.	11.89	11.37	11.37
		Max.	15.87	11.40	15.87
		Mean	14.06^{A,a}	11.39^{B,a}	13.17^{a}
	Green	*n*	6	2	8
		Min.	7.26	9.09	7.26
		Max.	14.39	10.50	14.39
		Mean	11.90^{A,a}	9.80^{B,a}	11.37^{a}
		Total mean	12.76^{A}	10.59^{A}	12.14
Linoleic acid (%)	Light green	*n*	4	2	6
		Min.	34.48	36.11	34.48
		Max.	36.97	37.53	37.53
		Mean	35.84^{A,a}	36.82^{A,b}	36.16^{a}
	Green	*n*	6	2	8
		Min.	33.28	39.83	3.28
		Max.	41.06	40.39	41.06
		Mean	37.13^{A,a}	40.11^{A,a}	37.88^{a}
		Total mean	36.62^{A}	38.46^{A}	37.14
Linolenic acid (%)	Light green	*n*	4	2	6
		Min.	14.93	17.28	14.93
		Max.	19.69	17.52	19.69
		Mean	16.37^{A,a}	17.40^{A,a}	16.72^{a}
	Green	*n*	6	2	8
		Min.	15.63	15.15	15.15
		Max.	18.87	16.32	18.87
		Mean	17.24^{A,a}	15.74^{A,a}	18.86^{a}
		Total mean	16.89^{A}	16.57^{A}	16.80
TSFA (%)	Light green	*n*	4	2	6
		Min.	33.19	33.79	33.19
		Max.	34.43	35.00	35.00

		Mean	33.73^{A,a}	34.39^{A,a}	33.95^{a}
	Green	*n*	6	2	8
		Min.	32.78	33.35	32.78
		Max.	35.58	35.37	35.58
		Mean	33.73^{A,a}	34.36^{A,a}	33.89^{a}
		Total mean	33.73^{A}	34.38^{A}	33.91
PUFA (%)	Light green	*n*	4	2	6
		Min.	50.78	53.63	50.78
		Max.	54.17	54.81	54.17
		Mean	52.21^{A,a}	54.22^{A,a}	52.88^{a}
	Green	*n*	6	2	8
		Min.	51.68	55.54	51.68
		Max.	58.11	56.15	58.11
		Mean	54.37^{A,a}	55.84^{A,a}	54.74^{a}
		Total mean	53.51^{A}	55.03^{A}	53.94
TUFA (%)	Light green	*n*	4	2	6
		Min.	65.57	65.00	65.00
		Max.	66.81	66.21	66.81
		Mean	66.27^{A,a}	65.61^{A,a}	66.05^{a}
	Green	*n*	6	2	8
		Min.	64.42	64.63	64.42
		Max.	67.22	66.65	67.22
		Mean	66.27^{A,a}	65.64^{A,a}	66.11^{a}
		Total mean	66.27^{A}	65.62^{A}	66.09
TPC (mg GAE/g)	Light green	*n*	4	2	6
		Min.	3.99	4.43	3.99
		Max.	5.69	5.29	5.69
		Mean	4.69^{A,a}	4.86^{A,b}	4.95^{b}
	Green	*n*	6	2	8
		Min.	4.84	7.00	4.84
		Max.	5.83	7.86	7.86
		Mean	5.26^{B,a}	7.43^{A,a}	6.11^{a}
		Total mean	4.92^{B}	6.15^{A}	5.35
TTC (mg CE/g)	Light green	*n*	4	2	6
		Min.	36.94	58.82	36.94
		Max.	58.59	67.38	67.38
		Mean	46.43^{B,a}	63.10^{A,a}	55.41^{a}
	Green	*n*	6	2	8
		Min.	39.69	44.90	39.69
		Max.	49.65	90.76	90.76
		Mean	43.87^{B,a}	67.83^{A,a}	55.00^{a}
		Total mean	44.94^{B}	65.47^{A}	50.77
TSC (mg DE/g)	Light green	*n*	4	2	6
		Min.	35.73	37.83	35.73
		Max.	56.58	61.80	37.83
		Mean	46.32^{A,a}	49.82^{A,a}	49.12^{a}
	Green	*n*	6	2	8
		Min.	31.80	51.84	31.80
		Max.	54.98	62.52	62.52

		Mean	41.75^{A,a}	57.18^{A,a}	48.06^{a}
		Total mean	43.69^{A}	53.50^{A}	46.41
DPPH (mg AAE/g)	Light green	*n*	4	2	6
		Min.	1.86	3.14	1.86
		Max.	2.85	4.86	3.14
		Mean	2.41^{B,a}	4.00^{A,a}	3.40^{b}
	Green	*n*	6	2	8
		Min.	2.26	4.32	2.26
		Max.	3.94	9.51	9.51
		Mean	3.11^{B,a}	6.92^{A,a}	4.76^{a}
		Total mean	2.70^{B}	5.46^{A}	3.58
ABTS (mg TE/g)	Light green	*n*	4	2	6
		Min.	7.09	8.47	7.09
		Max.	8.00	10.05	10.05
		Mean	7.41^{A,a}	9.26^{A,b}	8.78^{b}
	Green	*n*	6	2	8
		Min.	6.05	11.29	6.05
		Max.	8.39	16.76	16.76
		Mean	7.12^{B,a}	14.02^{A,a}	9.98^{a}
		Total mean	7.12^{B}	11.64^{A}	8.50
RP (mg AAE/g)	Light green	*n*	4	2	6
		Min.	3.33	4.75	3.33
		Max.	4.44	6.55	6.55
		Mean	4.05^{A,a}	5.65^{A,b}	5.11^{b}
	Green	*n*	6	2	8
		Min.	2.70	6.92	2.70
		Max.	4.45	9.48	9.48
		Mean	3.69^{B,a}	8.20^{A,a}	5.61^{a}
		Total mean	3.79^{B}	6.93^{A}	4.72

Different superscript capital letters in a row represent significantly different means within a category ($p < 0.05$). Different superscript small letters in a column represent significantly different means within a category ($p < 0.05$). ABTS: ABTS$^{\bullet+}$ scavenging activity, DPPH: DPPH$^{\bullet}$ scavenging activity, PUAF: total polyunsaturated fatty acid, RP: reducing power, TSFA: total saturated fatty acid, TUFA: total unsaturated fatty acid, TPC: total phenolic content, TSC: total saponin content, TTC: total tannin content.

Table S3. Analysis of variance data on the effects of pod color and seed color variations on the analyzed parameters in yardlong beans and contributions of the analyzed parameters to the variance observed in the principal component analysis (PCA).

		Factors			PCA contribution (%)	
Parameters	Components	Pod color (P)	Seed color (S)	P × S	Comp. 1	Comp. 2
Nutritional values	Crude protein	NS	NS	NS	0.00	18.70
	Total fat	NS	NS	NS	1.88	3.12
	Crude fiber	NS	NS	NS	1.22	0.50
	Dietary fiber	NS	NS	**	2.04	3.99
	Vitamin C	NS	NS	NS	1.64	0.16
Fatty acids	Palmitic acid	NS	NS	NS	2.04	20.91
	Stearic acid	NS	NS	NS	0.01	0.26
	Oleic acid	NS	NS	NS	7.02	4.61
	Linoleic acid	NS	NS	NS	6.65	0.04
	Linolenic acid	NS	NS	NS	0.30	1.30
	TSFA	NS	NS	NS	1.85	16.19
	PUFA	NS	NS	NS	1.85	16.20
	TUFA	NS	NS	NS	5.39	0.32
Total secondary metabolites	Total phenol	**	**	***	10.89	1.02
	Total tannin	NS	*	NS	8.41	4.21
	Total saponin	NS	NS	NS	8.42	0.72
Antioxidant activities	DPPH	*	*	*	12.80	3.70
	ABTS	**	**	***	14.08	2.45
	RP	**	**	***	13.51	1.60

ABTS: $ABTS^{•+}$ scavenging activity, DPPH: $DPPH^{•}$ scavenging activity, PUFA: total polyunsaturated fatty ac-id, RP: reducing power, TSFA: total saturated fatty acid, TUFA: total unsaturated fatty acid. ^{NS}Not significant, $^{*}p < 0.05$, $^{**}p < 0.01$, $^{***}p < 0.001$.
